# Morphometric magnetic resonance imaging study of the quadriceps tendon in hemodialysis patients: comparison with non-dialyzed controls

**DOI:** 10.1590/0100-3984.2021.0144

**Published:** 2022

**Authors:** Luis Marcelo de Azevedo Malta, Jocemir Ronaldo Lugon, Alair Augusto Sarmet Moreira Damas dos Santos, Leonardo Martins Machado

**Affiliations:** 1 School of Medicine, Universidade Federal Fluminense (UFF), Niterói, RJ, Brazil.

**Keywords:** Tendons/pathology, Magnetic resonance imaging, Renal insufficiency, chronic/complications, Renal dialysis, Risk factors, Hyperparathyroidism, Tendões/patologia, Ressonância magnética, Insuficiência renal crônica/complicações, Diálise renal, Fatores de risco, Hiperparatireoidismo

## Abstract

**Objective:**

To evaluate the knees of individuals with renal failure who are on hemodialysis, using magnetic resonance imaging (MRI), comparing them with those of a group of individuals with normal renal function.

**Materials and Methods:**

This was a cross-sectional, observational, controlled study conducted between August 2018 and February 2020. The cases consisted of 15 patients who had been on hemodialysis for ≥ 5 years and did not have a quadriceps tendon rupture. The controls consisted of 15 individuals with normal renal function who were matched (1:1) to the cases for sex, age, and physical activity level. The subjects in both groups underwent MRI of the right knee only.

**Results:**

The mean ages of the cases and controls were 50 ± 15 years and 49 ± 14 years, respectively. The median time on hemodialysis was 11 years (range, 10-14 years). Serum levels of parathyroid hormone, ferritin, alkaline phosphatase, phosphorus, and creatinine were higher among the cases than among the controls, whereas serum albumin and hemoglobin were lower (*p* < 0.05 for all). The MRI study showed a hyperintense signal in the quadriceps tendon in 11 of the cases and in three of the controls (*p* = 0.009). Knee joint effusion was observed in nine of the cases and in three of the controls (*p* < 0.05). The thickness, length, and width of the tendon did not differ between the groups. A hyperintense signal in the tendon was not associated with the time on hemodialysis; nor with the levels of intact parathyroid hormone, hemoglobin, or alkaline phosphatase.

**Conclusion:**

Patients on chronic hemodialysis, even those without a tendon rupture, show a hyperintense signal in the quadriceps tendon on MRI.

## Abstract (pt)

**Objetivo:**

Avaliar joelhos de indivíduos com falência renal em hemodiálise por meio de ressonância magnética, em comparação com um grupo controle sem doença renal crônica.

**Materiais e Métodos:**

Estudo transversal, observacional, controlado, realizado entre agosto/2018 e fevereiro/2020. Os 15 casos consistiram de pacientes com cinco anos ou mais em hemodiálise, sem ruptura do tendão do quadríceps. Os 15 controles, sem doença renal crônica, foram pareados (1:1) por sexo, idade e nível de atividade física.

**Resultados:**

A média de idade dos casos foi 50 ± 15 anos e a dos controles, 49 ± 14 anos. A mediana do tempo em hemodiálise foi 11 anos (variação: 10-14 anos). Nos casos, os níveis séricos de paratormônio, ferritina, fosfatase alcalina, fósforo e creatinina estavam mais altos e os de albumina e hemoglobina, mais baixos (*p* < 0,05). Hipersinal no tendão foi demonstrado em 11 casos e três controles (*p* = 0,009). Derrame articular foi observado em nove casos e três controles (*p* < 0,05). A espessura, o comprimento e a largura do tendão não diferiram entre os grupos. Hipersinal no tendão do quadríceps não mostrou associação com o tempo em hemodiálise ou com os níveis de paratormônio intacto, hemoglobina e fosfatase alcalina.

**Conclusão:**

Pacientes em hemodiálise sem episódios de ruptura já apresentam hipersinal no tendão do quadríceps nas imagens de ressonância magnética.

## INTRODUCTION

The quadriceps tendon is an important element in the biomechanics of gait and plays a major role in locomotion. Rupture of the quadriceps tendon is rare, typically occurring, either traumatically or spontaneously, in individuals over 40 years of age^(1-3)^. Patients who suffer from systemic illnesses are more prone to spontaneous rupture of their tendons, which results from the lower resistance of the tendons to contractions of the muscles. There have been several reports of spontaneous ruptures associated with a variety of diseases, including kidney diseases^(4-6)^.

There have been many studies establishing an association between metabolic disorders and tendon ruptures, the most common such disorder being hyperparathyroidism^(5,7-9)^. Although we do not know what kind of changes renal failure imposes on the structure of the quadriceps tendon, the relatively high occurrence of rupture leads us to believe that, in this circumstance, its resistance is diminished. To our knowledge, there have been no studies evaluating the imaging characteristics of tendons in hemodialysis patients, which could reveal abnormalities before the rupture events occur.

The knee extensor mechanism can be evaluated with a variety of imaging methods. For the study of soft tissues, ultrasound and magnetic resonance imaging (MRI) are the preferred modalities, given that both offer good spatial resolution and provide details regarding the texture of tissues^(10,11)^. Although some authors advocate the use of ultrasound because it is cheaper and more accessible^(11,12)^, most studies point to the superiority of MRI in defining the structural aspects of the tendon and in diagnosing its injury^(10,13-19)^.

The objective of this study was to understand the structural aspects of quadriceps tendons in hemodialysis patients. To that end, we employed MRI to evaluate the knees of individuals with chronic kidney disease (CKD) undergoing hemodialysis, comparing the findings with those of individuals who had normal renal function.

## MATERIALS AND METHODS

This was a cross-sectional, observational, controlled study in which quadriceps tendon data obtained by MRI and the results of laboratory tests of blood specimens were evaluated in a convenience sample of individuals with CKD undergoing hemodialysis, as well as in a group of individuals with normal renal function who were matched to the cases in terms of sex, age (± 5 years), handedness, and physical activity level. The research ethics committee of our institution approved the study project (Reference no. 2 566 617). All participants gave written informed consent.

The criteria for inclusion in the case group were being right-handed, having CKD, and having been undergoing hemodialysis on a regular basis for at least 5 years. The control group was composed of an equal number of individuals, with normal renal function and no history of any kidney disease or other systemic diseases, recruited from among patients admitted to the orthopedics department of a university hospital.

For both groups, individuals with a history of quadriceps tendon rupture were excluded, as were those who had been diagnosed with metastatic cancer, AIDS, or locomotor disorders of the lower limbs, as well as those with hemiplegia, those who were pregnant, and those who had undergone amputation of one or both lower limbs (including below-knee amputation). We also excluded individuals who had previously used quinolone antibiotics, which are known to increase the risk of tendon ruptures^(20,21)^. Demographic and anthropometric data were obtained with a specific form. Physical activity levels were determined with the International Physical Activity Questionnaire-Short Form.

In all of the individuals (in both groups), only the right knees were evaluated. Each individual underwent unenhanced MRI of the knee, and the same examination protocol was used for every examination. All MRI scans were performed in a 1.5-T scanner (Signa HDx; GE Healthcare, Waukesha, WI, USA). We employed multiplanar T1-weighted sequences and multiplanar proton density-weighted sequences with fat suppression. A descriptive analysis of the morphometry of the tendons was performed, together with measurements of their length, width, and thickness.

Patients in the case group had already undergone routine laboratory tests, and the resulting biochemical data were collected from their medical records. We used the mean of the last three values recorded for each variable. For patients in the control group, blood samples were collected by upper limb venous puncture, near the date of the imaging examination, and were submitted to laboratory testing. In both groups, we evaluated the serum levels of creatinine, albumin, hemoglobin, alkaline phosphatase, calcium, phosphorus, intact parathyroid hormone (iPTH), and ferritin, as well as transferrin saturation and hepatitis C serology.

### Statistical analysis

Variables were tested for normality with the Kolmogorov-Smirnov test. Results were expressed as mean and standard deviation for variables with normal distribution or as median and interquartile range for variables with Gaussian distribution. For intergroup comparisons of continuous variables, we used unpaired t-tests or the Mann-Whitney test as appropriate. Frequencies were compared by using the chi-square test or Fisher’s exact test. All statistical analyses were performed using the Predictive Analytics Software package, version 18.0 (SPSS Inc., Chicago, IL, USA).

## RESULTS

Between August 2018 and February 2020, a total of 30 MRI scans of the knees were performed (one scan per participant) and biochemical data were collected. The general characteristics of the participants are summarized in Table 1. The case group comprised 15 patients (13 males and two females), with a mean age of 50 ± 15 years. The control group also comprised 15 patients, with the same gender distribution and with a mean age of 49 ± 14 years.

**Table 1:** General characteristics of the participants.

Variable	Cases (n = 15)	Controls (n = 15)	P
Male gender, n (%)	13 (86.6)	13 (86.6)	—
Age (years), mean ± SD	50 ± 15	49 ± 14	0.801
Weight (kg), mean ± SD	64.7 ± 10.8	80.6 ± 15	0.003
Height (m), mean ± SD	1.66 ± 0.10	1.71 ± 0.11	0.223
Body mass index (kg/m^2^), mean ± SD	23.3 ± 3.1	27.2 ± 2.7	0.001
Primary renal disease, n (%)			
Diabetes mellitus	1 (6.6)	N/A	—
Hypertension	5 (33.3)	N/A	—
FSGS	2 (13.3)	N/A	—
Obstructive uropathy	1 (6.6)	N/A	—
Adult PKD	1 (6.6)	N/A	—
Unidentified	5 (33.3)	N/A	—
Time on hemodialysis (years), median (interquartile range)	11 (10-14)	N/A	—

SD, standard deviation; FSGS, focal segmental glomerulosclerosis; PKD, polycystic kidney disease; N/A, not applicable.

In the case group, the median time on hemodialysis was 11 years (interquartile range, 10-14 years). The causes of CKD were diabetes mellitus, in one patient; hypertension, in five; focal segmental glomerulosclerosis, in two; obstructive uropathy, in one; and adult polycystic kidney disease, in one. In the other five patients, the cause of CKD was not identified. Although the mean height was similar between the two groups, the mean weight was significantly higher in the control group, as was the body mass index.

The results of the laboratory tests are shown, by group, in Table 2. The mean serum levels of creatinine, ferritin, alkaline phosphatase, phosphorus, and iPTH were all significantly higher in the case group than in the control group (*p* < 0.05 for all). In contrast, the mean serum albumin level and the mean hemoglobin level were lower in the case group (*p* < 0.05 for both).

**Table 2:** Laboratory test results.

Variable	Cases (n = 15)	Controls (n = 15)	P
Positive serology for HCV, n (%)	1 (6.6)	0 (0)	N/A
Albumin (g/dL), median (IQR)	3.9 (3.7-4.1)	4.2 (4.0-4.5)	0.026
Creatinine (mg/dL), mean ± SD	11.2 ± 1.7	0.85 ± 0.1	< 0.001
Transferrin saturation (%), mean ± SD	32.5 ± 12.5	28 ± 9.5	0.284
Ferritin (µg/dL), median (IQR)	549 (338-684)	365 (168-514)	0.033
Calcium (mg/dL), mean ± SD	9.0 ± 0.6	9.3 ± 0.3	0.194
Phosphate (mg/dL), mean ± SD	4.6 ± 0.7	3.5 ± 0.5	< 0.001
Alkaline phosphatase (IU/L), median (IQR)	134 (88-364)	73 (57-96)	0.002
iPTH (pg/mL), median (IQR)	584 (283-1603)	40 (24.7-47.1)	< 0.001
Hemoglobin (g/dL), mean ± SD	10.7 ± 1.4	13.8 ± 1.6	< 0.001

HCV, hepatitis C virus; IQR, interquartile range; SD, standard deviation; N/A, not applicable.

**Figure 1:**
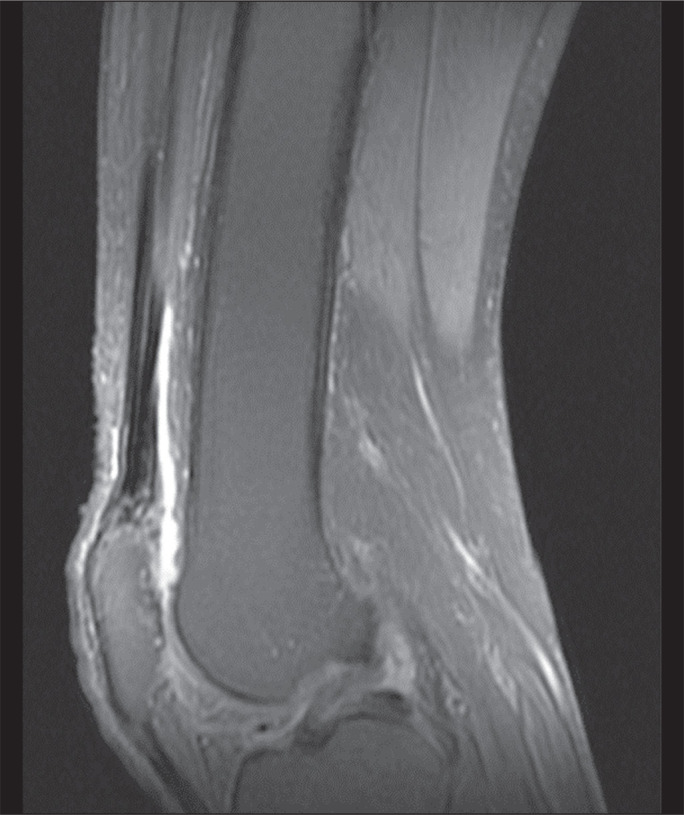
MRI of the knee, showing a hyperintense signal in the distal third of the quadriceps tendon.

**Figure 2:**
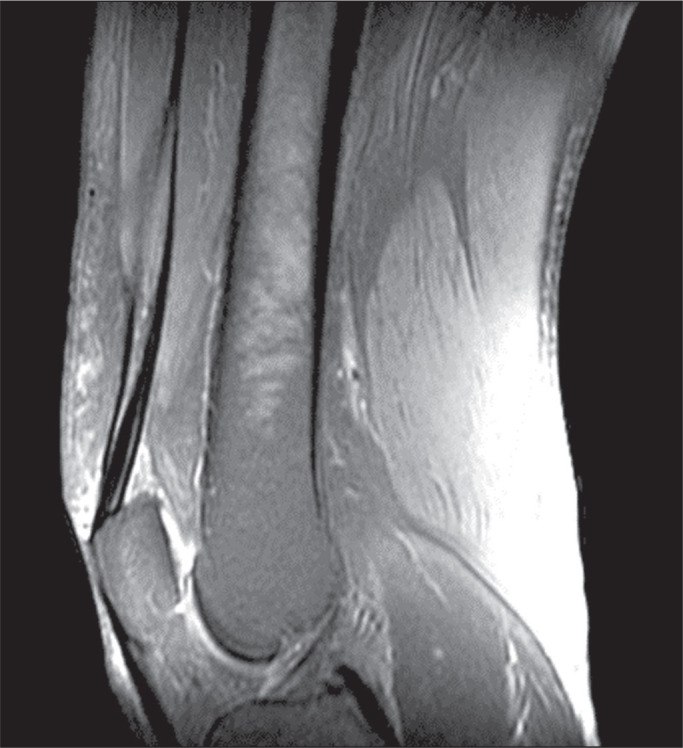
MRI of the knee, showing a diffuse hyperintense signal in the quadriceps tendon.

**Figure 3:**
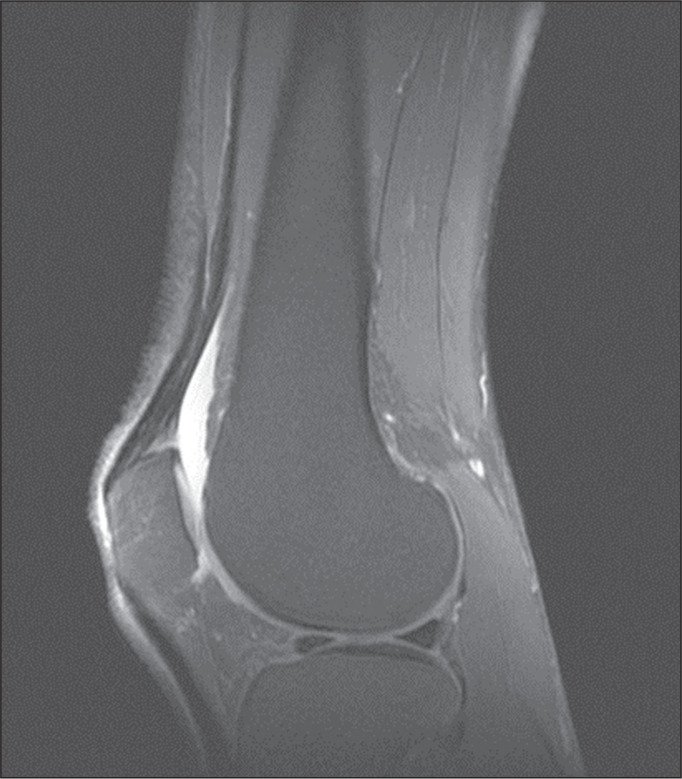
MRI of the knee, showing joint effusion.

**Figure 4:**
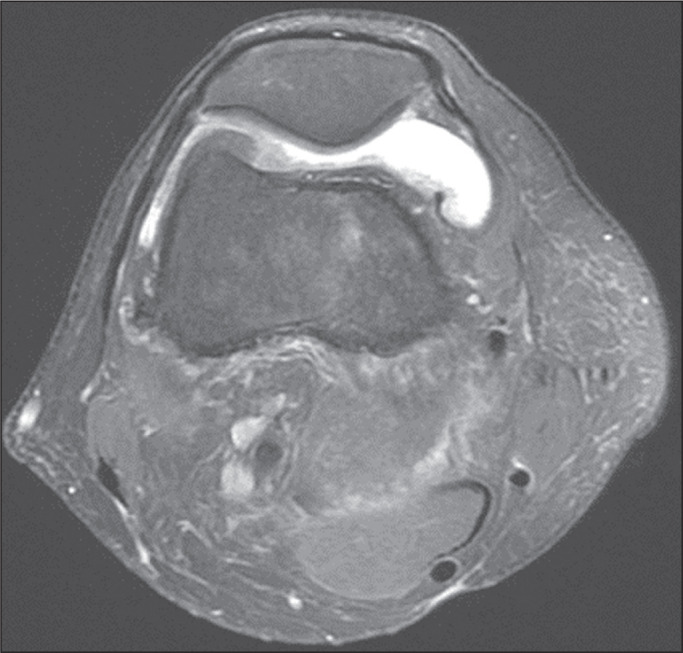
MRI of the knee, showing joint effusion.

The results of the morphometric analysis of the quadriceps tendons and the dimensions of those tendons are shown in Table 3. As can be seen in Figures 1 and 2, MRI showed a hyperintense signal in the quadriceps tendon in 11 of the subjects in the case group, compared with only three of those in the control group (*p* = 0.009). Likewise, knee joint effusion was seen in nine of the case group subjects and in three of the control group subjects (*p* < 0.05), as shown in Figures 3 and 4. However, the two groups were comparable in terms of the mean thickness, length, and width of the quadriceps tendon. In addition, signs of a partial quadriceps tendon tear were observed in three individuals (two in the case group and one in the control group), although the difference between the two groups was not statistically significant.

**Table 3:** Analysis of the MRI aspects of the knee.

Aspect	Cases (n = 15)	Controls (n = 15)	P
Quadriceps tendon dimensions, mean ± SD			
Thickness (cm)	0.81 ± 0.16	0.77 ± 0.16	0.448
Length (cm)	15.4 ± 1.12	15.1 ± 2.13	0.560
Width (cm)	2.3 ± 0.42	2.2 ± 0.45	0.420
Hyperintense signal, n (%)	11 (73.3)	3 (20.0)	0.009
Partial tear, n (%)	2 (13.3)	1 (6.7)	0.500
Joint effusion, n (%)	9 (60.0)	3 (20.0)	0.030

We attempted to determine whether a hyperintense signal in the quadriceps tendon was associated with some of the most relevant dichotomous variables (Table 4). We found that it was in fact not associated with the time on hemodialysis; nor was it associated with the levels of alkaline phosphatase, iPTH, or hemoglobin.

**Table 4:** Associations that a hyperintense signal in the quadriceps tendon showed with relevant parameters in hemodialysis patients.

Parameter	Cases with a hyperintense signal in the quadriceps tendon (n = 11)	P
Time on hemodialysis, n (%)		
≤ 10 years	3 (27.3)	0.235
> 10 years	8 (72.7)	
iPTH, n (%)		
≤ 600 pg/mL	5 (45.5)	0.569
> 600 pg/mL	6 (54.5)	
Hemoglobin, n (%)		
≤ 10 g/dL	3 (27.3)	0.235
> 10 g/dL	8 (72.7)	
Alkaline phosphatase, n (%)		
≤ 134 IU/L	5 (45.5)	0.569
> 134 IU/L	6 (54.5)	

The localization and the imaging aspect of the hyperintense signal in the quadriceps tendon varied across subjects. The most commonly affected portion of the tendon was the distal third, which showed a hyperintense signal in eight subjects (seven in the case group and one in the control group). In the remaining six examinations in which a hyperintense signal was detected (four in the case group and two in the control group), the signal was diffuse or was located in the middle or upper third of the tendon. Among the 11 case group subjects in whom a hyperintense signal was detected, the signal was considered irregular in three and homogeneous in eight.

Bone changes were detected on MRI in five of the subjects in the case group, lesions in the femoral diaphysis being detected in four of those five subjects. The findings were suggestive of bone infarction in two of those four subjects and of bone marrow reconversion in the other two. In the remaining patient, an alteration in the bone trabeculae, suggestive of a fracture, was noted at the upper pole of the patella.

## DISCUSSION

The quadriceps muscle tendon is a central structure in gait, and disturbances in its continuity can result in a number of limitations. Although there is a clear association between secondary hyperparathyroidism and tendon rupture^(4,22-25)^, there have been, to our knowledge, no studies evaluating the intrinsic characteristics of tendons prior to such an event. In the present study, we have described the aspects of quadriceps tendons in hemodialysis patients (cases), evaluating their dimensions and imaging characteristics on MRI, comparing them with those of healthy individuals (controls), and analyzing biochemical variables in both groups.

Our case group consisted mostly of men, although in a proportion higher than that reported for the dialysis population in Brazil^(26)^. The secondary hyperparathyroidism in our hemodialysis patients, as evidenced by the high levels of iPTH and alkaline phosphatase, is consistent with data in the literature related to quadriceps tendon injuries in patients with CKD^(4,22-24,27,28)^. The mean serum albumin and mean hemoglobin levels were lower in our case group than in our control group, whereas the mean serum levels of ferritin, phosphorus, and creatinine were higher. That is in keeping with our findings in a previous study of this topic^(28)^.

It is of note that the mean weight and body mass index were significantly lower in our case group than in our control group, as has been reported in other studies evaluating those parameters in patients with CKD^(29-31)^. However, despite the fact that the mechanical demand on anatomical structures should be greater in heavier individuals, we found that the imaging changes demonstrated in the tendons of our case group subjects were not affected by that statistically significant difference, the most compromised tendons being observed in the lighter individuals.

When examining the MRI acquisitions, we found that the sagittal slices offered the most detailed view of the signal intensity in the quadriceps tendon and its trilaminar aspect, as has been described in previous reports^(13,14,32-34)^. In addition, the mean dimensions of the tendons evaluated in our study seem to be in agreement with data in the literature^(34)^, despite the scarcity of studies describing such measures.

Analyzing the structural aspects of the quadriceps tendons on MRI, we found that signal changes (especially hyperintensity) were more common in our case group subjects. Despite the lack of studies similar to ours, normal tendons are typically described as having a hypointense signal on MRI^(13,18,34)^. In addition, studies evaluating quadriceps tendon injuries have demonstrated a hyperintense signal at the site of injury^(15,19)^. The signal abnormalities observed in our sample were predominantly in the distal third of the tendon. Although the patients evaluated in our study had not had a tendon rupture, that is also in keeping with data in the literature, which indicate that such events occur most commonly at the tendon-bone interface^(25,35,36)^, which corresponds to the distal third of the tendon.

When we considered only the hemodialysis patients in whom there were signal alterations in the tendons, we detected no effect of hemodialysis time on the appearance of such alterations, and that lack of association might be due to the small sample size. A number of previous studies have reported that prolonged time on hemodialysis is associated with a higher incidence of tendon ruptures^(25,27,35)^, because biochemical changes resulting from the underlying disease can lead to progressive weakening of a tendon, primarily close to its insertion^(27,35)^.

Although it was not the main focus of our study, knee joint effusion was significantly more common among the hemodialysis patients than among the subjects with normal renal function. Other authors have identified an association between chronic hemodialysis and amyloidosis, reporting findings similar to ours^(37,38)^.

Our study has some limitations. A more representative sample could have increased the validity of our findings, especially regarding the alterations seen on imaging. However, there have been very few studies evaluating the parameters defined in our study. To our knowledge, there have been no studies performing a systematic (controlled or uncontrolled) assessment of the morphology of the quadriceps tendon in patients with CKD who are on hemodialysis, which makes our study unique.

In conclusion, we were able to demonstrate that patients on chronic hemodialysis, even those with no history of tendon rupture, show a hyperintense signal in the quadriceps tendon on MRI.

## References

[1] Ilan DI, Tejwani N, Keschner M (2003). Quadriceps tendon rupture. J Am Acad Orthop Surg.

[2] Chiu M, Forman ES (2010). Bilateral quadriceps tendon rupture: a rare finding in a healthy
man after minimal trauma. Orthopedics.

[3] Siwek CW, Rao JP (1981). Ruptures of the extensor mechanism of the knee
joint. J Bone Joint Surg Am.

[4] Jones N, Kjellstrand CM (1996). Spontaneous tendon ruptures in patients on chronic
dialysis. Am J Kidney Dis.

[5] Levy M, Seelenfreund M, Maor P (1971). Bilateral spontaneous and simultaneous rupture of the quadriceps
tendon in gout. J Bone Joint Surg Br.

[6] Lombardi LJ, Cleri DJ, Epstein E (1995). Bilateral spontaneous quadriceps tendon rupture in a patient with
renal failure. Orthopedics.

[7] Cirincione RJ, Baker BE (1975). Tendon ruptures with secondary hyperparathyroidism. A case
report. J Bone Joint Surg Am.

[8] Preston FS, Adicoff A (1962). Hyperparathyroidism with avulsion of three major tendons. Report
of a case. N Engl J Med.

[9] Shiota E, Tsuchiya K, Yamaoka K (2002). Spontaneous major tendon ruptures in patients receiving long-term
hemodialysis. Clin Orthop Relat Res.

[10] Perfitt JS, Petrie MJ, Blundell CM (2014). Acute quadriceps tendon rupture: a pragmatic approach to
diagnostic imaging. Eur J Orthop Surg Traumatol.

[11] Warden SJ, Kiss ZS, Malara FA (2007). Comparative accuracy of magnetic resonance imaging and
ultrasonography in confirming clinically diagnosed patellar
tendinopathy. Am J Sports Med.

[12] Bianchi S, Poletti PA, Martinoli C (2006). Ultrasound appearance of tendon tears. Part 2: lower extremity
and myotendinous tears. Skeletal Radiol.

[13] Soudry M, Lanir A, Angel D (1986). Anatomy of the normal knee as seen by magnetic resonance
imaging. J Bone Joint Surg Br.

[14] Abram SGF, Sharma AD, Arvind C (2012). A traumatic quadriceps tendon tear associated with calcific
tendonitis. BMJ Case Rep.

[15] Hodgson RJ, O’Connor PJ, Grainger AJ (2012). Tendon and ligament imaging. Br J Radiol.

[16] Swamy GN, Nanjayan SK, Yallappa S (2012). Is ultrasound diagnosis reliable in acute extensor tendon
injuries of the knee?. Acta Orthop Belg.

[17] Saragaglia D, Pison A, Rubens-Duval B (2013). Acute and old ruptures of the extensor apparatus of the knee in
adults (excluding knee replacement). Orthop Traumatol Surg Res.

[18] Yablon CM, Pai D, Dong Q (2014). Magnetic resonance imaging of the extensor
mechanism. Magn Reson Imaging Clin N Am.

[19] McMahon CJ, Ramappa A, Lee K (2017). The extensor mechanism: imaging and intervention. Semin Musculoskelet Radiol.

[20] Marsen TA, Pollok M, Baldamus CA (1999). Spontaneous tendon rupture after ofloxacin treatment in renal
transplant recipients on high-dose corticosteroids. Am J Kidney Dis.

[21] Muzi F, Gravante E, Tati E (2007). Fluoroquinolones-induced tendinitis and tendon rupture in kidney
transplant recipients: 2 cases and a review of the
literature. Transplant Proc.

[22] Lotem M, Bernheim J, Conforty B (1978). Spontaneous rupture of tendons. A complication of hemodialyzed
patients treated for renal failure. Nephron.

[23] De Franco P, Varghese J, Brown WW (1994). Secondary hyperparathyroidism, and not beta 2-microglobulin
amyloid, as a cause of spontaneous tendon rupture in patients on chronic
hemodialysis. Am J Kidney Dis.

[24] Thaunat M, Gaudin P, Naret C (2006). Role of secondary hyperparathyroidism in spontaneous rupture of
the quadriceps tendon complicating chronic renal failure. Rheumatology (Oxford).

[25] Muratli HH, Çelebi L, Hapa O (2005). Simultaneous rupture of the quadriceps tendon and contralateral
patellar in a patient with chronic renal failure. J Orthop Sci.

[26] Sesso RCC, Lopes AA, Thomé FS (2012). Chronic dialysis in Brazil: report of the Brazilian dialysis
census, 2011. J Bras Nefrol.

[27] Carmo LPF, Oliveira RA, Abensur H (2010). Spontaneous bilateral rupture of quadriceps tendon: first case in
short daily hemodialysis. NDT Plus.

[28] Malta LMA, Gameiro VS, Sampaio EA (2014). Quadriceps tendon rupture in maintenance haemodialysis patients:
results of surgical treatment and analysis of risk factors. Injury.

[29] Pérez-Torres A, González Garcia ME, San José-Valiente B (2018). Protein energy wasting syndrome in advanced chronic kidney
disease: prevalence and specific clinical characteristics. Nefrologia (England Ed).

[30] Qureshi AR, Alvestrand A, Danielsson A (1998). Factors predicting malnutrition in hemodialysis patients: a
cross-sectional study. Kidney Int.

[31] Kalantar-Zadeh K, Kopple JD, Block G (2001). Association among SF36 quality of life measures and nutrition,
hospitalization, and mortality in hemodialysis. J Am Soc Nephrol.

[32] Chien A, Weaver JS, Kinne E (2020). Magnetic resonance imaging of the knee. Pol J Radiol.

[33] el-Khoury GY, Brandser EA, Saltzman CL (1994). MRI of tendon injuries. Iowa Orthop J.

[34] Yu JS, Petersilge C, Sartoris DJ (1994). MR imaging of injuries of the extensor mechanism of the
knee. Radiographics.

[35] Matokovic D, Matijasevic B, Petrić P (2010). A case report of spontaneous concurrent bilateral rupture of the
quadriceps tendons in a patient with chronic renal failure. Ther Apher Dial.

[36] Pei YC, Hsieh PC, Huang LZ (2011). Simultaneous bilateral quadriceps tendon rupture in a uremic
patient. Formosan Journal of Musculoskeletal Disorders.

[37] Kurer MH, Baillod RA, Madgwick JC. (1991). Musculoskeletal manifestations of amyloidosis. A review of 83 patients in haemodialysis for at least 10 years. J Bone
Joint Surg Br.

[38] Sigaux J, Abdelkefi I, Bardin T (2019). Tendon thickening in dialysis-related joint arthritis is due to
amyloid deposits at the surface of the tendon. Joint Bone Spine.

